# Surface Integrity in the Grinding of Hardened AISI D2 Steel

**DOI:** 10.3390/ma18040814

**Published:** 2025-02-13

**Authors:** Tao Zhang, Qianting Wang, Ningchang Wang, Lan Yan, Feng Jiang, Enlai Zhang, Wuhui Zhou, Hao Gao, Yong Wang

**Affiliations:** 1 College of Mechanical and Automotive Engineering, Xiamen University of Technology, Xiamen 361021, China; zhangtao.ime@hqu.edu.cn; 2 SINOMACH Intelligence Technology Co., Ltd., Guangzhou 510670, China; 3 State Key Laboratory of High-Performance Tools, Zhengzhou Research Institute for Abrasives & Grinding Co., Ltd., Zhengzhou 450001, China; wangningchang@126.com; 4 College of Mechanical Engineering and Automation, National Huaqiao University, Xiamen 361021, China; jiangfeng@hqu.edu.cn; 5 ZYNP Corporation, Mengzhou 454750, China; zouwuhui@hnzynp.com (W.Z.); wangyong@hnzynp.com (Y.W.); 6 School of Mechanical and Electrical Engineering, Sanming University, Sanming 365004, China; gaohaozynp@sohu.com

**Keywords:** surface integrity, roughness, subsurface microstructure, hardness, residual stress

## Abstract

The surface integrity of grinding has a significant influence on the service performance and life of machined parts. In this study, the influence of grinding parameters and the grit size of the grinding wheel on the surface integrity of the hardened steel, including the roughness, microstructure, and hardness of the subsurface and the residual stress of the ground surface, was comprehensively investigated, and the corresponding mechanisms were revealed. The results show that the roughness perpendicular to the grinding direction was significantly larger than that parallel to the grinding direction due to serious side flow, and increasing the grinding speed or reducing the grinding depth was beneficial for reducing the side flow and thus decreasing the roughness. It was found that the grinding temperature dominated the formation of a harder white layer and a softer black layer, and workpiece speed had the smallest effect on the transition of subsurface microstructure compared to grinding speed and depth. It was also found that the increase in workpiece speed, grinding depth, or grinding speed resulted in a transition from compressive to tensile residual stress or an increase in tensile residual stress, and that grinding wheels with finer grit tended to induce compressive residual stress. This study may help to improve surface integrity by optimizing grinding parameters or facilitating the selection of the optimal grinding wheel.

## 1. Introduction

Grinding is usually the last process in machining parts and requires high precision. Both machining precision and surface integrity are vital for grinding [1,2]. The machine tools, the dressing of the grinding wheel, the compensation of the program, etc., can guarantee machining precision. However, achieving high surface integrity for the machined surface is more difficult due to the multiple evaluations and complex mechanisms [3,4]. Surface integrity significantly affects the properties of friction pairs [5], fatigue resistance [6], and corrosion resistance [7], which directly determine the service performance and life of the machined parts. The surface integrity of machined surfaces has received more attention in recent years. Surface integrity is a broad definition that involves roughness, microstructure, hardness, and residual stress [8].

Compared with cutting, the achievement of lower roughness of the machined surface is more straightforward for grinding because it involves micrometer-scale microcutting of massive fine grit and minimal grit distances [9,10,11]. Optimal dressing, in which the grit is sharpened and the number of grits involved in the grinding is increased, is vital for reducing roughness [12]. Theoretically, the decrease in feed or grinding depth helps to reduce the roughness due to the reduction in undeformed chip thickness [13]. However, too small undeformed chip thicknesses may lead to significant side flow instead of chip formation during microcutting of grit, which worsens the roughness [14]. In general, increased grinding speed helps to reduce the roughness by decreasing the side flow [15].

The deterioration of the layer seems inevitable during grinding due to the high grinding temperature and extensive plastic deformation. During the grinding of metals with high hardness, a white layer, which is a type of deteriorated layer, is often formed [16,17,18]. The white layer has characteristics of high hardness, low ductility, etc. [19]. A large number of studies have shown that the white layer is composed of secondary quenched martensite, residual austenite, and carbide, and is often harder and more brittle than the substrate [20]. Guo et al. [21] found that the content of chemical elements (e.g., C, O, and Mn) in the white layer differs from that in the substrate. Unfortunately, the related mechanisms have not been determined. Xu et al. [22] found that the properties of materials also impact the white layer, and the thickness of the white layer increases with the increase in the content of C. The residual austenite helps with the formation of the white layer. Bill et al. [23] found that the dislocation energy of the material has an essential effect on the microstructure of the grinding-induced white layer.

Grinding-induced residual stress is caused by mechanical deformation, thermal deformation, and phase transformation [24,25,26]. For the mechanical deformation, cutting the grit with a big negative rake angle during the grinding process causes a skinny deformation layer, which leads to compressive residual stress on the ground surface and tensile residual stress in the subsurface. A smaller cutting depth of the grit tends to induce higher compressive residual stress in the ground surface [27]. With regard to thermal deformation, the high grinding temperature causes thermal deformation during the grinding process. The shrinkage of the surface caused by cooling after grinding is restricted by the subsurface, which results in the tensile residual stress of the ground surface and the compressive residual stress of the subsurface [28]. Regarding the phase transformation, the densities for different phases vary. Therefore, the phase transformation caused by high temperature and severe plastic deformation could introduce residual stress [29]. Recent studies showed that the surface is in a state of compressive residual stress when mechanical deformation is the dominant factor. In contrast, the tensile residual stress of the ground surface will be generated when thermal deformation is the dominant factor [30]. The residual stress will not only affect the corrosion resistance and service life of the parts but also warp the parts and affect their precision [31].

AISI D2 steel is the typical cold-working mold steel. Grinding is the most common process for the machining of hardening steels. The deterioration of surface integrity after grinding including the unexpected roughness deterioration, seriously deteriorated layer, and more considerable residual stress is a common problem, which will seriously worsen the service performance of the mold. Therefore, it is essential to study the surface integrity of the grinding surface to improve the life and performance of the manufactured mold. Studies about the grinding of AISI D2 steel have been carried out. The grinding ability of AISI D2 steel could be improved with ultrasonic vibration based on the comprehensive evaluation of grinding forces, force ratios, surface roughness, and morphology [32]. The effect of abrasive type and cooling mode on the fatigue resistance of the AISI D2 ground surface has been studied. It was found that the sol–gel abrasive wheel and cryogenic cooling mode significantly improved surface integrity and fatigue life compared with the combination of the Al_2_O_3_ abrasive wheel and soluble oil cooling mode [6]. The influence of nano-minimum-quantity lubrication on the surface roughness during grinding of AISI D2 steel has been studied. The results showed that the surface roughness was reduced by 77% when a grinding fluid of CuO nanopowder in colza base oil was used compared with pure oil [33]. The multiobjective optimization of grinding of AISI D2 steel with an Al_2_O_3_ wheel was implemented with the objective of optimizing surface quality, surface temperature, and normal force to obtain the optimal parameters [34]. A novel nitriding grinding has been proposed to improve the grindability and surface integrity of AISI D2 [35].

Although the grinding of AISI D2 steel has been extensively studied, comprehensive research about surface integrity in the grinding of AISI D2 steel is lacking. The study of surface integrity in the grinding of AISI D2 steel is essential to guide the improvement of the surface integrity of grinding for hardened AISI D2 steel, which has great application significance. In this study, the ground surface integrity of hardened AISI D2 steel, including the roughness and residual stress parallel and perpendicular to the grinding direction, and the subsurface microstructure are investigated experimentally. The effects of grinding parameters and the grit size of the grinding wheel on the surface integrity and the related mechanisms have been revealed.

## 2. Design of the Experiment

### 2.1. The Grinding Experiment

The grinding experiments were carried out on a CNC ultraprecision flat-surface grinder (type number: MGK7120-6; manufacturer: Zhejiang Hangji Co., Ltd., Hangzhou, China) with a feed precision of 0.1 μm, a maximum spindle power of 2.2 kW, and a maximum spindle rotation speed of 3000 rpm. Hardened AISI D2 steel with a hardness of HV715 was selected as the workpiece material. The chemical compositions and mechanical properties of the workpiece material are shown in Table 1 and Table 2, respectively. The dimensions of the sample for the grinding experiments were 8 mm × 15 mm × 20 mm, and the size of the grinding surface was 15 mm × 20 mm. The sample was held in place by a precise vice which was adsorbed on the magnetic worktable of the grinder during the grinding test. The longest edge of the specimen was along the grinding direction. White aluminum oxide grinding wheels with vitrified bonds (Zhengzhou Research Institute for Abrasives & Grinding Co., Ltd., Zhengzhou, China) were used for the grinding experiments. Grinding wheels with grain sizes of 60# (P200 × 20 × 32WA60L8V) and 80# (P200 × 20 × 32WA80L8V) were used. A HOCUT795 water-based emulsion (Quaker HOUGHTO Co., Ltd., Conshohocken, PA, USA) with a concentration of 4% was selected as the grinding fluid. The flow rate was set as 15 L/min. Grinding wheel dressing, using a diamond dressing pen, and pre-grinding were implemented before each group of grinding experiments to ensure the stable performance of the grinding wheels.

**Figure 1:**
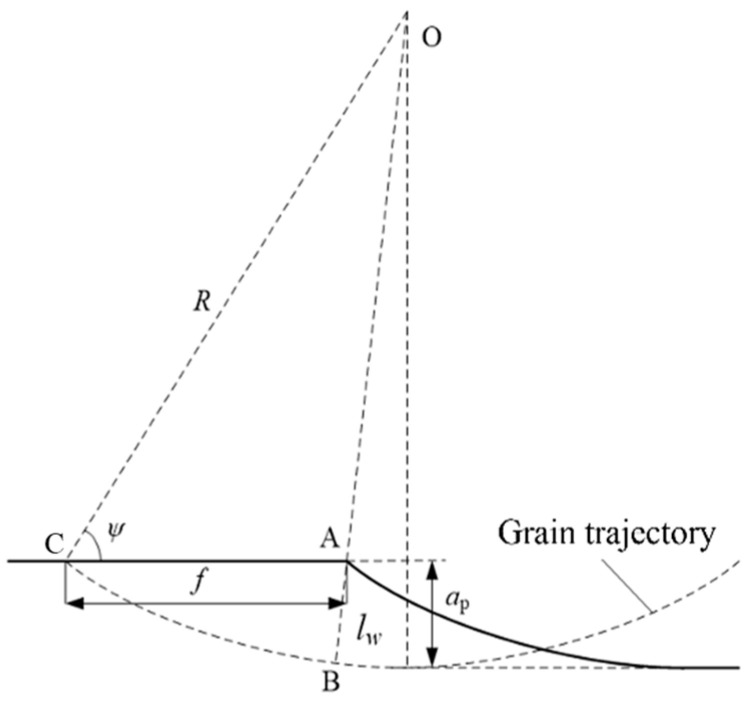
A sketch of undeformed chip thickness in surface grinding.

The grain trajectory of the surface grinding is presented in Figure 1, in which, *a_p_*, *l_w_*, *f*, and *R* are the grinding depth, undeformed chip thickness, feed per revolution, and diameter of the grinding wheel. *ψ* is the angle between *OC* and *AC*, where *C* is the highest contact point between the workpiece and grinding wheel and *O* is the center of the grinding wheel. In this study, the diameter of the grinding wheel *R* was 200 mm. According to Figure 1, *l_w_* described the actual cutting depth of the grain. Therefore, *l_w_* is also an important variable, as well as grinding speed and *a_p_*, which could be determined using the following equations.

(1) $$l_{w}=R-\sqrt{R^{2}+f^{2}-2Rf\cos\psi }$$

(2) $$\psi =\arcsin\left(1-\frac{a_{p}}{R}\right)$$

To comprehensively investigate the impact of the grinding speed, grinding depth, and undeformed chip thickness on the surface integrity of the ground surface, three groups of grinding experiments investigating a single factor were arranged, as shown in Table 3, Table 4 and Table 5.

### 2.2. The Detection of Surface Integrity

In this study, the roughness, microhardness, and microstructure of the subsurface, as well as the residual stress, were measured and analyzed to comprehensively study the surface integrity of the ground surface. A white light interferometer (Wkyo NT9300, Veeco Instruments Inc., Plainview, NY, USA) with a resolution of 0.1 nm was used to measure the surface roughness. The measurement area was 4.5 mm × 4.5 mm, and each measurement was repeated three times. The measurement areas were located along the central line with intervals of 6 mm, parallel to the specimen’s longest edge. The measured results were processed by high-pass filtering to exclude the effect of flatness error on the roughness evaluation. Then, the roughness of the ground surface was evaluated in the directions parallel and perpendicular to the grinding direction because of the anisotropy of the roughness of the ground surface. The average roughness of three measurement areas was calculated to evaluate the roughness of the ground surface in the directions parallel and perpendicular to the grinding direction. This study assessed the roughness using the arithmetic mean deviation of the profile *Ra*, the root mean square deviation of *Rq*, and the ten-point height of microscopic unflatness, *Rp*.

**Figure 2:**
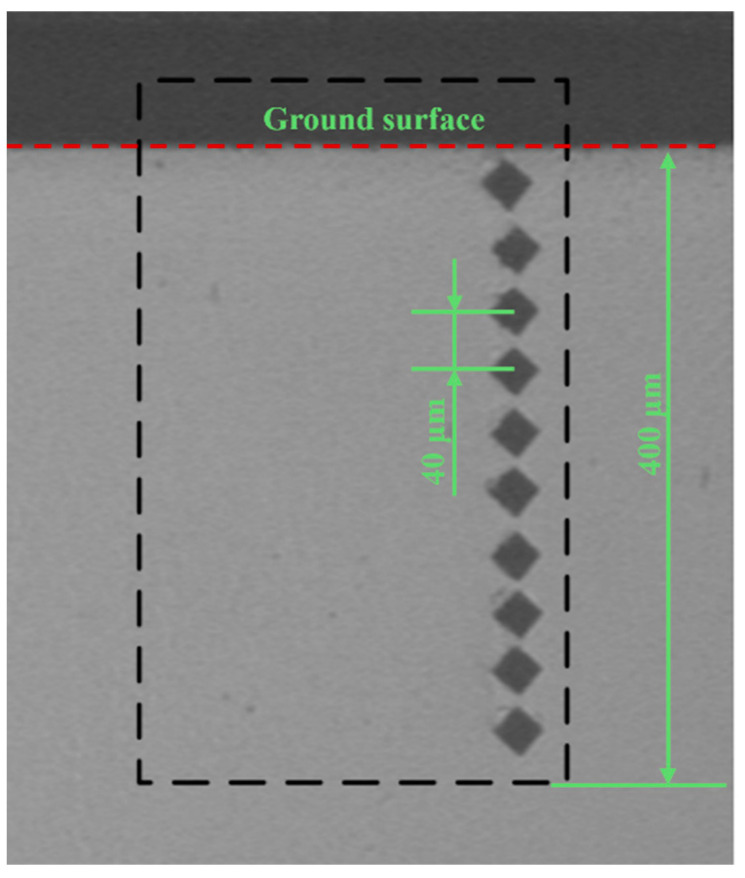
A sketch of the measurement of the microhardness of the subsurface.

Regarding the microhardness of the subsurface, after the grinding experiment, the specimens were cut perpendicular to the grinding direction by wire electrical discharge machining. The subsurface was ground and polished after the preparation of the cross-section. The microhardness was measured by the HV-1000 microhardness tester (Jinan Yihua tester Co., Ltd., Jinan, China) in compliance with the GB/T4340.1-2009 standard [36]. The microhardness tests’ loading and loading hold time were 100 gf and 5 s, respectively. The microhardness of the subsurface within the distance from the ground surface of 400 μm, with a measurement distance of 40 μm, was detected by a microhardness tester, as shown in Figure 2.

To observe the subsurface microstructure, a smooth subsurface was prepared using the same procedure mentioned above. Then, cross-sections were corroded by an etchant (5% HNO_3_ + 95% C_2_H_6_O) with a corrosion time of 30 s followed by cleaning and drying. Finally, the microstructure of the subsurface was observed by a metallurgical microscope (Olympus-GX41, Olympus Corporation, Tokyo, Japan).

**Figure 3:**
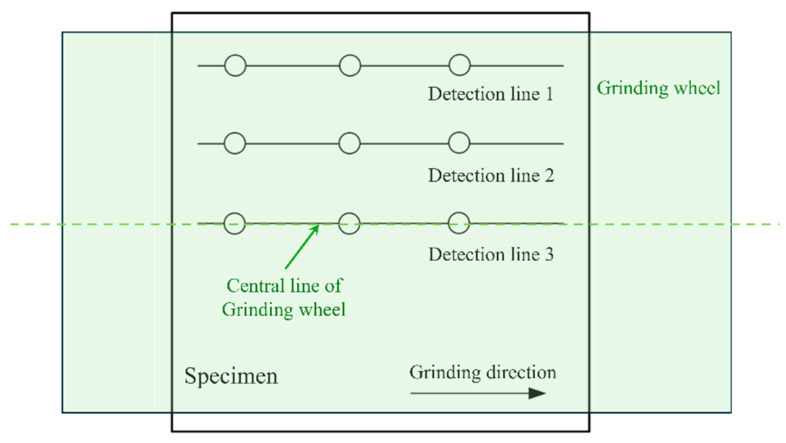
Sketch of the method for the detection of residual stress.

The residual stresses parallel and perpendicular to the grinding direction were measured by a S3000 residual stress tester (XSTRES Co., Ltd., Ravenhall, Australia), in which the residual stress was detected based on X-ray diffraction. The voltage, current, and exposure time were set to 30 kV, 6.7 mA, and 15 s, respectively. As shown in Figure 3, the grinding-induced residual stress of measuring points at different distances from the center line of the grinding wheel may be different. Therefore, the detection lines were arranged at various distances from the central line of the grinding wheel, and the residual stresses of three points located along the detection line were measured to calculate the average stress.

## 3. Results and Discussion

### 3.1. The Roughness of the Ground Surface

**Figure 4:**
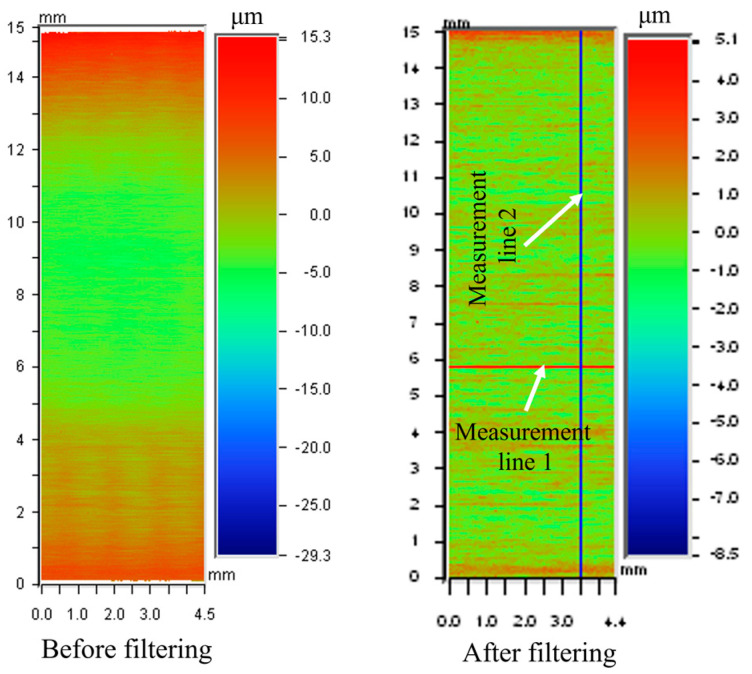
The morphology of the ground surface before and after filtering (60# grinding wheel; *V_w_*: 0.03 m/s; *a_p_*: 5 μm; *S*: 10.5 m/s).

The typical morphology of the ground surface is shown in Figure 4. A non-negligible shape error was found in the morphology of the ground surface before filtering. Therefore, high-pass filtering was implemented before the determination of roughness. The measurement lines used to obtain the roughness parallel and perpendicular to the grinding direction are presented in Figure 4.

**Figure 5:**
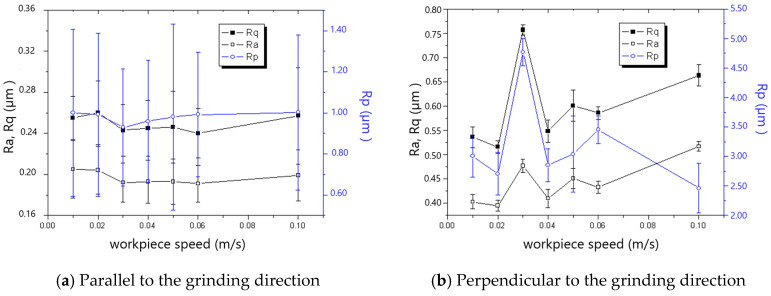
The effect of workpiece speed on roughness (60# grinding wheel; *a_p_*: 5 μm; *S*: 20.9 m/s).

**Figure 6:**
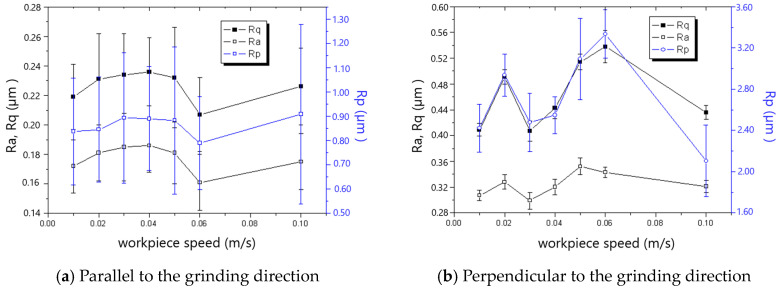
The effect of workpiece speed on roughness (80# grinding wheel; *a_p_*: 5 μm; *S*: 20.9 m/s).

**Figure 7:**
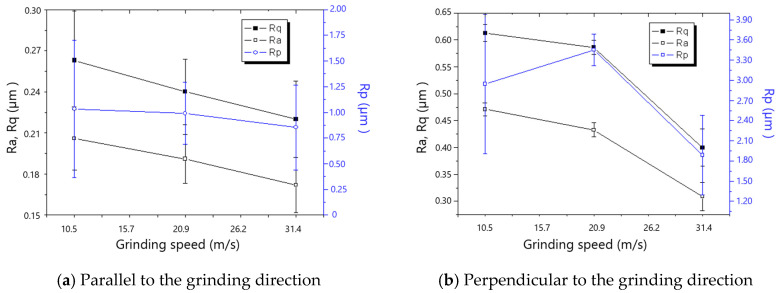
The effect of grinding speed on roughness (60# grinding wheel; *a_p_*: 5 μm; *t_c_*: 34.2 μm).

**Figure 8:**
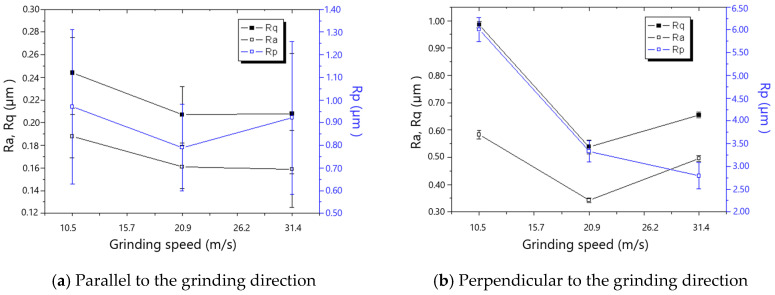
The effect of grinding speed on roughness (80# grinding wheel; *a_p_*: 5 μm; *t_c_*: 34.2 μm).

**Figure 9:**
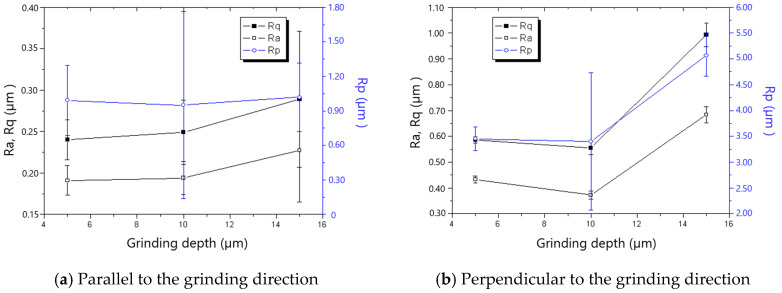
The effect of grinding depth on roughness (60# grinding wheel; *S*: 20.9 m/s; *t_c_*: 34.2 μm).

**Figure 10:**
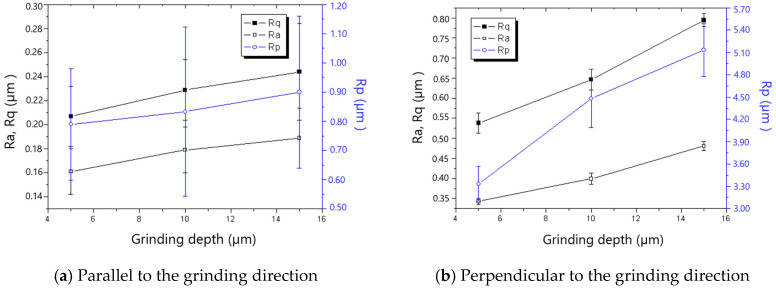
The effect of grinding depth on roughness (80# grinding wheel; *S*: 20.9 m/s; *t_c_*: 34.2 μm).

The measured roughness is shown in Figure 5, Figure 6, Figure 7, Figure 8, Figure 9 and Figure 10. It was found that the roughness parallel to the grinding direction was less than that perpendicular to the grinding direction. The material side flow was severe due to the minimal cutting depth of the grit, which significantly worsened the roughness perpendicular to the grinding direction. Additionally, a bigger grit size resulted in a more serious material side flow, contributing to the phenomenon that the roughness of the 60# grinding wheel was larger than that of the 80# grinding wheel.

In general, the roughness perpendicular to the grinding direction decreased with the decline in workpiece speed, as shown in Figure 5 and Figure 6. However, when the workpiece speed was 0.03 m/s and 0.02 m/s for the 60# grinding wheel and 80# grinding wheel, respectively, the roughness perpendicular to the grinding direction became abnormal. There is a critical difference in the cutting depth in the transition from cutting to ploughing, and as a result, most of the material in front of the grit flows sideways instead of forming a chip when the actual cutting depth is smaller than the critical cutting depth, leading to the remarkable rise in roughness perpendicular to the grinding direction. In addition, the necessary cutting depth grows with the increase in grit size. Therefore, the workpiece speed resulting in the appearance of abnormal roughness for the 60# grinding wheel is larger than that for the 80# grinding wheel. It could be concluded from the above analysis that the workpiece speed corresponding to the critical cutting depth resulting in the transition from cutting to ploughing should be avoided to improve the roughness in grinding. This viewpoint contradicts the general assumption that roughness can be enhanced as long as the worktable speed is reduced.

Figure 7 and Figure 8 show that roughness decreases as the grinding speed increases. This is because the presence of material in front of the grit tends to result in the formation of a chip, which reduces the side flow and then decreases the roughness, especially in the direction perpendicular to the grinding direction. As shown in Figure 9 and Figure 10, the roughness rises with the increase in grinding depth. The main reason is that a higher grinding temperature is induced by the large grinding depth, causing higher flowability and more side flow of the material in front of the grit, which results in the rise in roughness. The increase in roughness perpendicular to the grinding direction is faster than that parallel to the grinding direction with the increase in grinding depth.

### 3.2. The Microstructure and Hardness of the Subsurface

**Figure 11:**
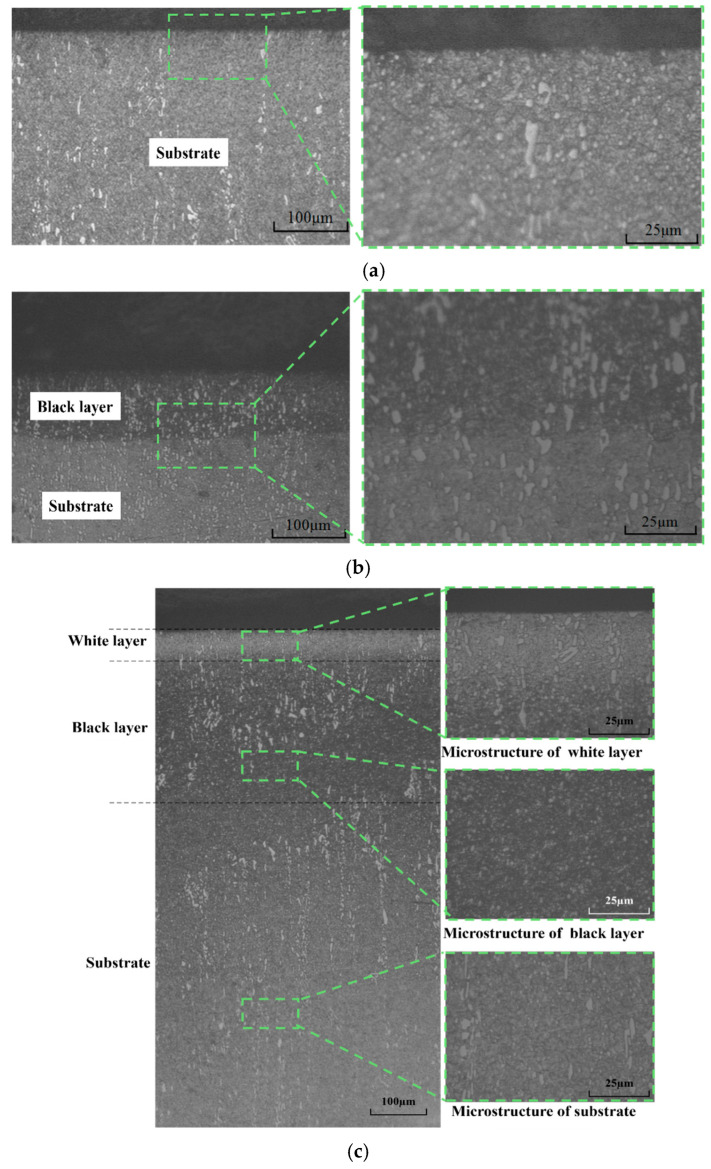
The typical microstructure of the ground subsurface. (**a**) The subsurface without the obvious transition of microstructure (60# grinding wheel; *f_w_*: 0.1 m/s; *a_p_*: 5 μm; *S*: 31.5 m/s). (**b**) The subsurface with a black layer (60# grinding wheel; *V_w_*: 0.02 m/s; *a_p_*: 15 μm; *S*: 20.9 m/s). (**c**) The subsurface with black and white layers (80# grinding wheel; *V_w_*: 0.047 m/s; *a_p_*: 15 μm; *S*: 20.9 m/s).

The observed three typical microstructures of the ground subsurface are shown in Figure 11. The grinding temperature is the dominant factor affecting the subsurface microstructure. When the grinding temperature is low (<400 °C), low-temperature tempering occurs on the subsurface, and the initial microstructure of the substrate transitions into tempered martensite with a lower hardness than the substrate. However, the transition of the microstructure cannot be observed, as shown in Figure 11a. When the grinding temperature is about 400 °C to 600 °C, tempered troostite appears because of moderate tempering, and a black layer could be found, as shown in Figure 11b. In addition, when the grinding temperature is about 600 °C to 1000 °C, the initial microstructure transitions into the harder tempered sorbite due to high-temperature tempering. The transitioned tempered sorbite also presents as a black layer, as shown in Figure 11b. The hardness of tempered troostite and tempered sorbite is usually lower than that of the substrate. When the grinding temperature is higher than the temperature required for α-γ transition, austenitizing will occur, and the harder martensite will form after rapid cooling because of the quench. The martensite, with an extremely small grain size and 20% greater hardness than the substrate, is white after etching, and thus is referred to as the white layer, as shown in Figure 11c.

**Figure 12:**
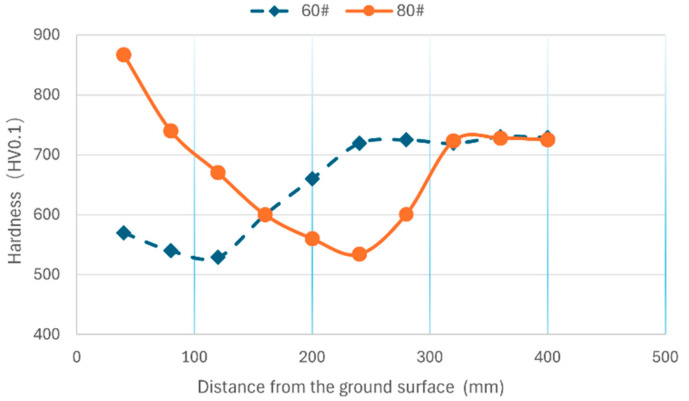
The microhardness of the subsurface (*V_w_*: 0.1 m/s; *a_p_*: 5 μm; *S*: 20.9 m/s).

The typical microhardnesses of the subsurface with different distances from the ground surface are shown in Figure 12. The measured hardness–distance curves for 60# and 80# grinding wheels are different because of the various phase transformations determined by the grinding temperature. As for the hardness–distance curve for the 80# grinding wheel, the higher grinding temperature resulting from the finer grit size induces the formation of a white layer with remarkably higher hardness compared to the substrate with an average hardness of about HV715. The hardness decreases with the increase in distance from the ground surface, and the lowest hardness of HV508.32 is achieved at a depth of about 250 μm. Then, the hardness rises with the increase in distance from the ground surface and reaches the same hardness as the substrate at a depth of about 350 μm. The hardness of the zone between the depth from approximately 100 μm to 350 μm is lower than that of the substrate because its microstructure is a black layer or tempered martensite. Regarding the subsurface hardness–distance curve for the 60# grinding wheel, the hardness for distances less than 200 μm is less than that of the substrate, which also results from the black layer or tempered martensite.

The subsurface microstructures and the corresponding maximum and minimum hardness for the designed grinding experiments were summarized, as shown in Table 6, Table 7 and Table 8. It could be found that the subsurface microstructure tends to transition as the grinding temperature rises due to the increases in grinding speed, grinding depth, and workpiece speed. However, the workpiece speed has the smallest effect on the transition of subsurface microstructure compared to grinding speed and grinding depth. This means that the combination of a reduced grinding depth and speed and an increased workpiece speed could achieve the same material removal rate and a smaller subsurface microstructure transition. It could also be found that the maximum hardness is significantly higher than that of the substrate as long as a white layer is formed, and that the minimum hardness is always lower than that of the substrate because of the black layer or tempered martensite.

### 3.3. The Residual Stress of the Ground Surface

**Figure 13:**
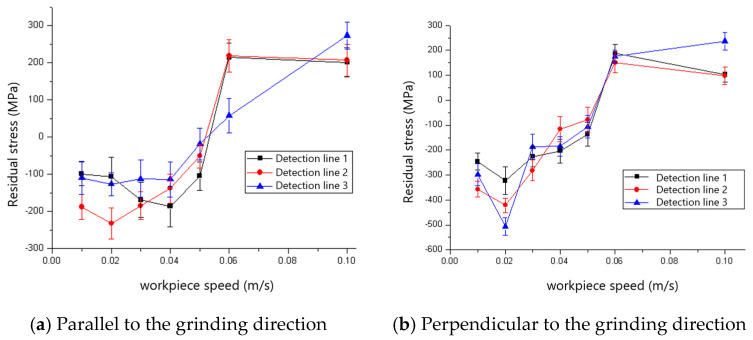
The effect of workpiece speed on residual stress (60# grinding wheel; *a_p_*: 5 μm; *S*: 20.9 m/s).

**Figure 14:**
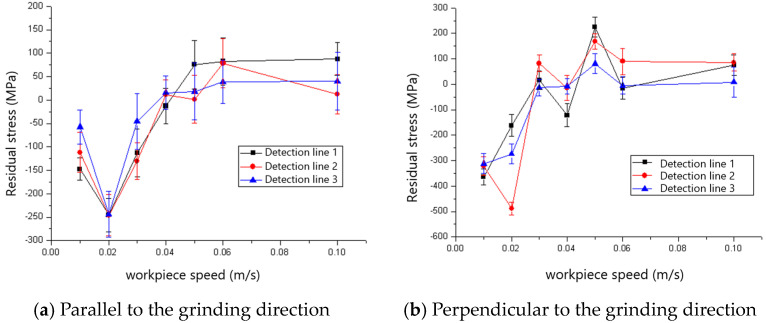
The effect of workpiece speed on residual stress (80# grinding wheel; *a_p_*: 5 μm; *S*: 20.9 m/s).

The effect of workpiece speed on the residual stress of the ground surface is shown in Figure 13 and Figure 14. The residual stresses both parallel and perpendicular to the grinding direction could be found to shift from compressive residual stress to tensile residual stress as the workpiece speed increased. The critical workpiece speeds for the compressive-to-tensile residual stress transition are 0.04 m/s and 0.05 m/s for the 60# and 80# grinding wheels, respectively. The cutting depth of grit increases with the rise in workpiece speed according to the experimental design shown in Table 1, which leads to increased grit cutting of the workpiece instead of scratching or ploughing. Then, mechanical deformation-inducing compressive residual stress becomes the non-dominant factor, which results in the state transition of the residual stress shown in Figure 13 and Figure 14. It is found that the maximum compressive stress appears when the grinding speed is 0.02 m/s for both the 60# and 80# grinding wheels. It is also found that the maximum compressive stress that appears perpendicular to the grinding direction is larger than that which is parallel to the grinding direction, both for 60# and 80# grinding wheels, because of the serious side flow caused by the big tip radius of the grit. By comparing Figure 13 and Figure 14, it is clear that the maximum tensile residual stress for the 80# grinding wheel is smaller than that of the 60# grinding wheel because more grits cut the workpiece instead of sliding or ploughing for the 80# grinding wheel with sharper grit.

**Figure 15:**
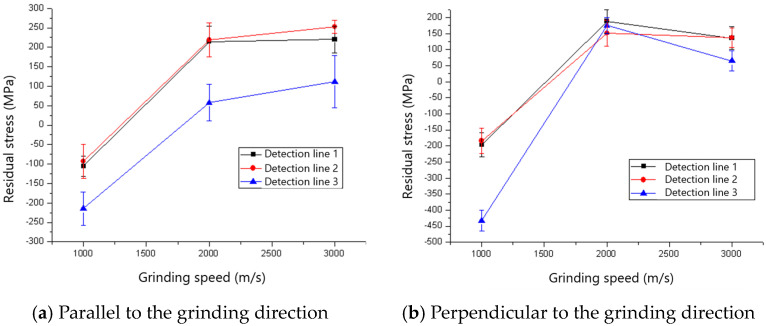
The effect of grinding speed on residual stress (60# grinding wheel; *a_p_*: 5 μm; *t_c_*: 34.2 μm).

**Figure 16:**
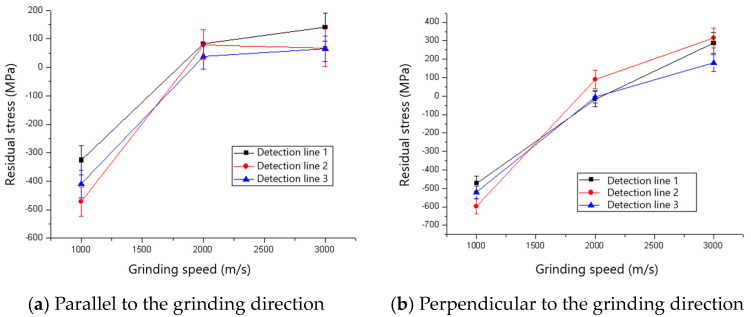
The effect of grinding speed on residual stress (80# grinding wheel; *a_p_*: 5 μm; *t_c_*: 34.2 μm).

The changes in residual stresses parallel and perpendicular to the grinding direction of the ground surface with the increase in grinding speed are shown in Figure 15 and Figure 16. The compressive residual stresses transition into tensile residual stresses with the increases in grinding speed, and the critical grinding speeds for the transition are 1500 rev/min and 2000 rev/min for 60# and 80# grinding wheels, respectively. The reason for the transition is that the increased grinding temperature as a result of the rise in grinding speed results in the thermal deformation-inducing tensile residual stress becoming the dominant factor.

**Figure 17:**
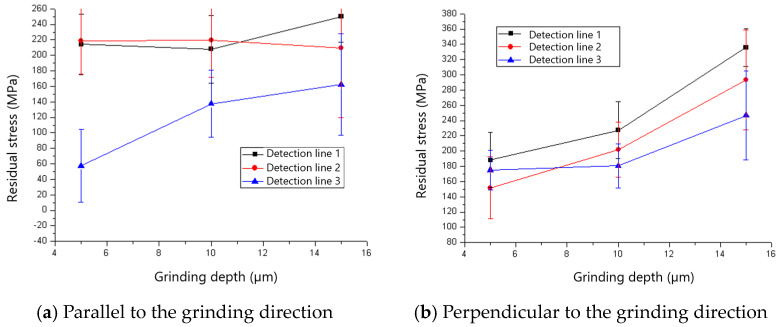
The effect of grinding depth on residual stress (60# grinding wheel; *S*: 20.9 m/s; *t_c_*: 34.2 μm).

**Figure 18:**
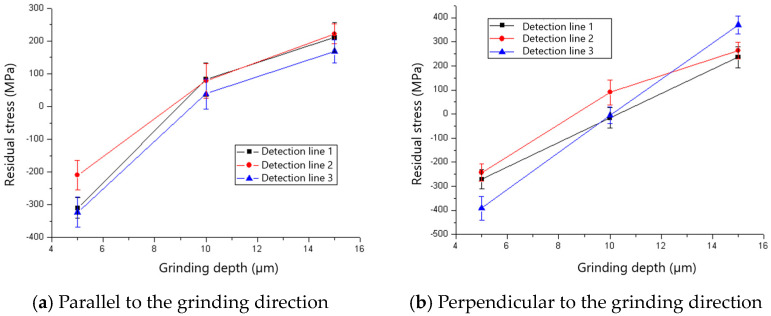
The effect of grinding depth on residual stress (80# grinding wheel; *S*: 20.9 m/s; *t_c_*: 34.2 μm).

The residual stresses parallel and perpendicular to the grinding direction for various grinding depths when the 60# grinding wheel was used are shown in Figure 17. It could be found that the ground surfaces are in a state of tensile residual stress which tends to increase with the rise in grinding depth. The reason for the rise in tensile residual stress is that the increased grinding temperature as aa result of the rise in grinding depth induces higher tensile residual stress. The influence of grinding depth on the residual stress for an 80# grinding wheel is shown in Figure 18. There is a transition from compressive to tensile residual stress as the grinding depth increases, and the critical grinding depth is about 10 μm. The change and transition of the residual stress shown in Figure 18 is also dominated by thermal deformation.

Comparing the residual stress of 60# and 80# grinding wheels, it could be found that the 80# grinding wheel tends to induce compressive residual stress. The reason for this phenomenon is that the increased grit scratching or ploughing of the workpiece that occurs instead of cutting due to the higher grit density of the 80# grinding wheel results in more serious mechanical deformation, which induces compressive stress. 

## 4. Conclusions and Outlook

In this study, the ground surface integrity, including the roughness and residual stress parallel and perpendicular to the grinding direction, and the microstructure of the subsurface of hardened AISI D2 steel are investigated experimentally using 60# and 80# ceramic bonded alumina grinding wheels. The effect of grinding parameters and the grit size of the grinding wheel on the ground surface integrity and the corresponding mechanisms was revealed. The main conclusions are summarized as follows:

- The serious material side flow results in the roughness perpendicular to the grinding direction being significantly larger than that parallel to the grinding direction. Sometimes, a too small undeformed chip thickness may lead to unexpected side flow, remarkably worsening the roughness. Regarding the grinding parameters assessed in this study, increased grinding speed or reduced grinding depth is beneficial in reducing the side flow and decreasing the ground surface’s roughness. The roughness of the 60# grinding wheel is larger than that of the 80# grinding wheel.
- Three typical ground subsurfaces were identified, including a subsurface without an obvious transition of microstructure, a subsurface with a black layer, and a subsurface with white and black layers. The grinding temperature is the dominant factor determining the formation of white and black layers. It has been found that the workpiece speed has the smallest effect on the transition of subsurface microstructure compared to grinding speed and depth. The maximum hardness of the subsurface is significantly higher than that of the substrate as long as a white layer is formed, and the minimum hardness of the subsurface is always lower than that of the substrate because of the black layer or tempered martensite.
- Within the grinding parameters used in this study, the residual stresses shifted from compressive residual stress to tensile residual stress as a result of the mechanical deformation becoming the non-dominant factor as the workpiece speed increased. Increasing the grinding speed or depth will improve the grinding temperature which in turn leads to a transition from compressive to tensile residual stress or a rise in tensile residual stress. The 80# grinding wheel tended to induce greater compressive residual stress compared with the 60# grinding wheel.
- The formation mechanisms of surface integrity are extremely complex because of the numerous influencing factors. Several mechanisms have not yet been revealed just based on experiments. In the future, numerical simulations of grinding or single-grit scratching should be implemented to clarify the mechanisms further.

## Figures and Tables

**Table 1:** The chemical composition of AISI D2 steel.

Element	C	Si	Mn	P	S	Cr	Ni	Mo	Cu	V	Fe
wt%	1.59	0.38	0.35	0.024	0.015	11.68	0.36	0.67	0.03	0.39	Balance

**Table 2:** The mechanical properties of AISI D2 steel.

Hardness	Elastic Modulus	Yield Stress	Ultimate Strength	Fracture Toughness *K_IC_*	Poisson Ratio
61 HRC	209.9 GPa	2050 MPa	2200 MPa	7.94 MN/m^2/3^	0.285

**Table 3:** The experiment design for different undeformed chip thicknesses.

Experiment No.	Workpiece Speed *V_w_* (m/s)	Grinding Depth *a_p_* (μm)	Grinding Speed *S* (m/s)	Undeformed Chip Thickness *t_c_* (μm)
1	0.01	5	20.9	3.45
2	0.02	5	20.9	7.8
3	0.03	5	20.9	13.05
4	0.04	5	20.9	19.2
5	0.05	5	20.9	26.25
6	0.06	5	20.9	34.2
7	0.1	5	20.9	75.01

**Table 4:** The experiment design for different grinding speeds.

Experiment No.	Workpiece Speed *V_w_* (m/s)	Grinding Depth *a_p_* (μm)	Grinding Speed *S* (m/s)	Undeformed Chip Thickness *t_c_* (μm)
1	0.09	5	31.4	34.2
2	0.06	5	20.9	34.2
3	0.03	5	10.5	34.2

**Table 5:** The experiment design for different grinding depths.

Experiment No.	Workpiece Speed *V_w_* (m/s)	Grinding Depth *a_p_* (μm)	Grinding Speed *S* (m/s)	Undeformed Chip Thickness *t_c_* (μm)
1	0.06	5	20.9	34.2
2	0.052	10	20.9	34.2
3	0.047	15	20.9	34.2

**Table 6:** Summary of the subsurface microstructure for the experiments listed in Table 1.

Grinding Wheel	Experiment No.	Without Obvious Transition	Black Layer Depth (μm)	White Layer Depth (μm)	Maximum Microhardness	Minimum Microhardness
60#	1	**√**	N/A	N/A	709.66	575.79
	2	**√**	N/A	N/A	699.87	608.94
	3	**√**	N/A	N/A	713.98	618.27
	4	**√**	N/A	N/A	737.69	581.43
	5	N/A	170	N/A	745.31	591.56
	6	**√**	N/A	N/A	722.39	562.19
	7	N/A	200	N/A	730.14	529.81
80#	1	**√**	N/A	N/A	711.41	579.37
	2	**√**	N/A	N/A	706.53	598.41
	3	**√**	N/A	N/A	678.98	608.81
	4	**√**	N/A	N/A	754.39	563.29
	5	**√**	N/A	N/A	734.56	585.61
	6	N/A	180	N/A	801.36	521.73
	7	N/A	255	65	867.61	534.50

**Table 7:** Summary of the subsurface microstructure for the experiments listed in Table 2.

Grinding Wheel	Experiment No.	Without Obvious Transition	Black Layer Depth (μm)	White Layer Depth (μm)	Maximum Microhardness	Minimum Microhardness
60#	1	N/A	230	N/A	716.54	526.54
	2	**√**	N/A	N/A	722.39	592.19
	3	**√**	N/A	N/A	698.76	600.45
80#	1	N/A	210	50	819.17	510.45
	2	**√**	N/A	N/A	801.36	531.73
	3	**√**	N/A	N/A	722.41	556.65

**Table 8:** Summary of the subsurface microstructure for the experiments listed in Table 3.

Grinding Wheel	Experiment No.	Without Obvious Transition	Black Layer Depth (μm)	White Layer Depth (μm)	Maximum Microhardness	Minimum Microhardness
60#	1	**√**	N/A	N/A	722.39	613.19
	2	N/A	170	N/A	754.18	560.34
	3	N/A	150	30	829.19	539.81
80#	1	**√**	N/A	N/A	801.36	589.73
	2	N/A	200	50	888.31	564.72
	3	N/A	300	60	894.56	530.68

## Data Availability

The original contributions presented in this study are included in the article. Further inquiries can be directed to the corresponding authors.
